# G-quadruplex profiling using single-cell CUT&Tag offers insight into the epigenetic landscape of the developing mouse brain

**DOI:** 10.1186/s13059-026-04339-w

**Published:** 2026-11-13

**Authors:** Jing Lyu, Szabolcs Hetey, Mehmet Ali Öztürk, Gonçalo Castelo-Branco, Marek Bartosovic, Simon J. Elsässer

**Affiliations:** 1 Science for Life Laboratory, Department of Medical Biochemistry and Biophysics, Division of Genome Biology, Karolinska Institutet, Tomtebodavägen 23, 17165 Stockholm, Sweden; 2 Ming Wai Lau Centre for Reparative Medicine, Karolinska Institutet, Stockholm Node, Solnavägen 9, Stockholm, 17165 Sweden; 3 Institute for Biology, Universität Freiburg, Schänzlestrasse 1, 79104 Freiburg, Germany; 4 Center for Integrative Biological Signalling Studies (CIBSS), University of Freiburg, Schänzlestrasse 18, 79104 Freiburg, Germany; 5 Department of Medical Biochemistry and Biophysics, Laboratory of Molecular Neurobiology, Karolinska Institutet, Solnavägen 9, Stockholm, 17165 Sweden; 6 Department of Biochemistry and Biophysics, Stockholm University, Svante Arrhenius Väg 16, 10405 Stockholm, Sweden

## Abstract

**Background:**

G-quadruplexes (G4s) are non-canonical DNA structures that have gained increasing attention for their potential roles in gene regulation and role in neurodegenerative diseases and cancer. Despite their biological significance, G4 structures have not been studied systematically across tissues and cell types.

**Results:**

In this study, we employ G4 single-cell CUT&Tag (G4 scCUT&Tag) to characterize G4 landscapes in postnatal mouse brain cells, leveraging single-cell analytical approaches commonly used in scRNA-Seq and scATAC-Seq datasets. Using conventional single-cell omics workflows to process and explore our data, we distinguish distinct cell lineages based on G4 heterogeneity and establish that a subset of lineage-specific genes show unique promoter G4s. Multi-omics integration with scRNA-Seq gene expression profiles, using both a covariance-based technique (canonical correlation analysis) and a transfer learning-based approach, enables more accurate cell-type annotation. These integrations not only reveal correlations between G4 signal and gene expression, but demonstrate that G4 scCUT&Tag enables detailed examination of G4 heterogeneity in complex tissues and supports integrative analysis of G4 profiles with other omics layers, offering new insights into the epigenomic landscapes of the developing brain.

**Conclusions:**

Our findings establish G4 scCUT&Tag as a framework for investigating the cellular heterogeneity of G4 landscapes in complex tissues and provide a basis for exploring their relationships with gene regulation and other layers of chromatin organization.

**Supplementary Information:**

The online version contains supplementary material available at https://doi.org/10.1186/s13059-026-04339-w.

## Background

G-quadruplexes (G4s) are four-stranded secondary structures that form in guanine-rich DNA and RNA. Their building block is the G-tetrad, a planar arrangement of four guanines held together by Hoogsteen hydrogen bonding; two or more stacked tetrads coordinated by a central monovalent cation, such as potassium in physiological condition, constitute a G4 structure [1, 2]. Sequences able to fold into such structures typically contain four runs of three or more consecutive guanines separated by short loops [3]. Polymerase-stalling assays performed on purified genomic DNA (G4-seq) have mapped several hundred thousand sequences with G4-forming potential (Putative G-Quadruplex-Forming Sequence, PQS) in the human genome and comparable numbers in mouse and other vertebrates [4, 5]. Only a small fraction of this potential is realized in chromatin, with state-of-the-art mapping techniques in cells typically detecting around 10–30 thousand folded G4s, corresponding to approximately 1–3% of the sequences capable of forming G4 structures in vitro. Open, accessible chromatin context is considered a prerequisite, but not sole determinant of whether a given sequence forms a G4 in a particular cellular context. Consistent with this a large fraction of G4 associates with G-rich promoters of constitutively expressed genes [6, 7], as well as at telomeres and other loci containing guanine-rich repeats [8–10]. Beyond promoters, G4s are also enriched at active cis-regulatory elements and frequently coincide with transcription factor occupancy [7, 11, 12]. Moreover, G4 formation can differ between cell types and has been associated with cell-type-specific transcriptional programs [13]. These observations raise the possibility that facultative G4s may contribute to, or serve as markers of, cell-type-specific cis-regulatory activity. Hence, it is crucial to understand cell-type specific G4 landscapes and their context-dependent regulation.

G4s may have functional consequences at multiple levels. In transcriptional regulation, G4s can act as barriers at certain promoters to repress transcription [14], while at other loci they are thought to help maintain open chromatin and gene activity [7, 15]. Persistent G4s ahead of replication forks can impede fork progression and represent a recognized source of genome instability, which cells mitigate through G4-resolving helicases, including BLM, WRN and DHX36 [16, 17]. This has driven interest in G4-stabilizing ligands as potential anticancer agents and has increasingly implicated G4 dysregulation in aging and neurodegenerative diseases [16, 18–21]. G4-stabilizing ligands elicit distinct responses across neurons, astrocytes and microglia [22], while G4-forming repeat expansions, such as the GGGGCC hexanucleotide expansion in *C9orf72*, are causally linked to amyotrophic lateral sclerosis and frontotemporal dementia [23]. More broadly, G4s have been proposed to play regulatory roles in neural gene expression [17, 24–26].

Defining where G4s fold in vivo has relied on mapping technologies, each capturing a distinct aspect of G4 biology. G4-seq measures the intrinsic G4-forming potential of purified genomic DNA and therefore does not account for chromatin context and cell-type specific regulation [4, 5]. By contrast, G4 ChIP-seq, using the structure-specific BG4 antibody, maps folded G4s in cells, but requires formaldehyde crosslinking, sonication-based chromatin fragmentation and approximately 10^7^ cells per experiment [7, 27]. We previously adapted CUT&Tag for G4 mapping [28, 29], enabling detection of folded G4s under native, non-crosslinked conditions from approximately 10^5^ cells while achieving substantially higher signal-to-noise than G4 ChIP-seq. These improvements bring G4 profiling within reach of samples with limited cellular input, including primary material.

Single-cell CUT&Tag has enabled profiling of histone modifications and transcription factors across individual cells in complex tissues [30, 31], providing a framework for extending chromatin profiling to G4 structures. More recently, G4 scCUT&Tag has been demonstrated in a mixture of two human cancer cell lines [32], establishing the feasibility of measuring G4 landscapes at single-cell resolution.

A key unresolved question is whether G4s can be robustly profiled in primary, heterogeneous tissues and whether their folding patterns reflect cell identity. The postnatal mouse brain provides a compelling system in which to address these questions. G4 biology has clear relevance to neurological disease, while the diverse cell populations of the brain span markedly different proliferative states, ranging from dividing oligodendrocyte progenitor cells to mature oligodendrocytes and neurons. Importantly, high-quality single-cell RNA-seq and single-cell CUT&Tag reference datasets are available from the same tissue and developmental stage [30, 33], providing independent benchmarks against which cell identities inferred solely from G4 landscapes can be evaluated.

## Results

### scCUT&Tag mapping of G-quadruplexes allows for distinguishing specific cell populations

**Fig. 1:**
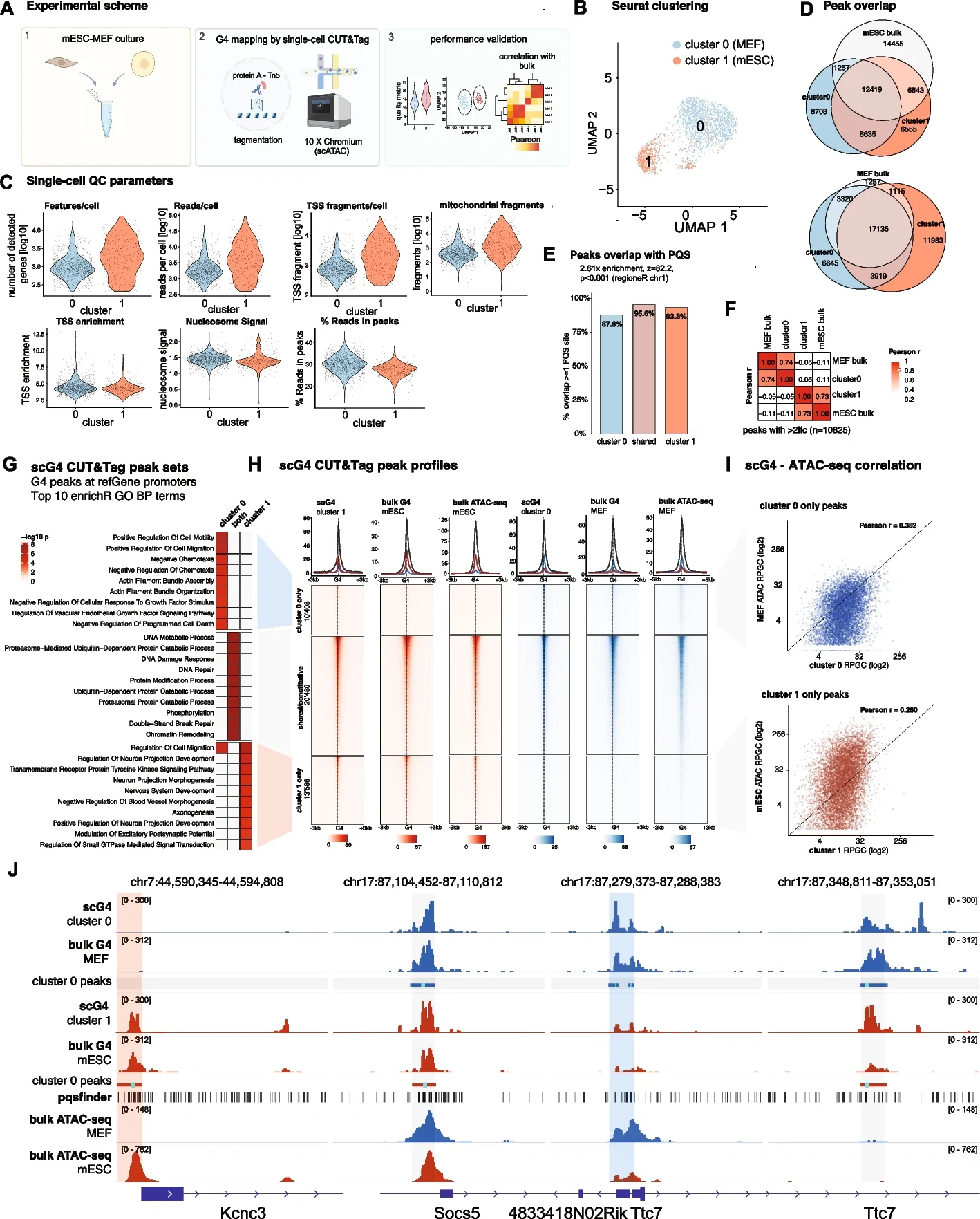
G4 profiling by scCUT&Tag is capable of separating different cell types. **A**. Schematic view of the mESC-MEF G4 scCUT&Tag experimental design. A mix of two defined cell populations, mouse embryonic stem cells (mESC) and mouse embryonic fibroblasts (MEF) was used to assess the resolution of G4 scCUT&Tag. Nuclei were isolated, incubated with a G4 antibody (BG4), tagmented using a protein A-Tn5 fusion, and processed following the 10 × Chromium scATAC-seq protocol. Read count matrices were processed, quality checked, normalized, and explored. **B**. UMAP followed by clustering performed on the mESC-MEF G4 scCUT&Tag returns two different clusters. The resolution parameter of SNN-based clustering was set to 0.1. Cells are colored according to Seurat clusters. **C**. Violin plots showing the quality measures computed by Seurat. All the metrics were computed by Signac. **D**. Venn diagrams show peak overlaps among Seurat cluster 0, cluster 1, and mESC bulk G4 CUT&Tag, as well as among Seurat cluster 0, cluster 1, and MEF bulk G4 CUT&Tag. **E**. Bargraph showing PQS overlap with scG4 peaks per cluster, results of regioneR permutation test is given for combined peaks. **F**. Correlation heatmap of differential (|lfc| > 2) peaks between cluster 0 and cluster 1 (n = 10,825), showing Pearson correlation between scG4 cluster pseudobulk signal and bulk mESC/MEF bulk G4 datasets. **G**. EnrichR GO analysis of cluster-specific gene sets (cl0 = 1,452, shared = 8,953, cl1 = 2,318). Top 10 terms shown with *p* value. Cluster 0-only, shared, cluster 1-only scG4 peaks from D were overlapped with promoters ± 2 kb to yield cluster-specific gene sets. **H**. Heatmaps of scG4 pseudobulk normalized signal, corresponding bulk G4 and ATAC signals, at unique and overlapping peaks as defined in D. all signals are normalized by Reads Per Genomic Content (RPGC). **I**. Pearson correlation between (top) scG4 pseudobulk scG4 signal cluster 0 and MEF ATAC-seq signal (r = 0.382) at cluster 0-only G4 peaks (*n* = 10,407), as well as (bottom) scG4 signal cluster 1 and mESC ATAC-seq signal (r = 0.260) at cluster 1-only G4 peaks (*n* = 13,576). **J**.) Representative genome browser tracks of scG4 pseudobulk signals, bulk G4, and matched bulk ATAC. PQS regions are shown with pqsfinder score > 20. Examples of G4 peaks with (*Kcnc3*, *Ttc7* promoter) and without accessibility (*Ttc7* gene body) are shown. All signals are RPGC normalized

In order to map G4s at the single-cell level we developed a highly sensitive centrifugation-based variant of our bulk G4 CUT&Tag [28] with a previously established scCUT&Tag protocol based on the droplet-based 10 × Genomics scATAC kit [30] (Fig. 1A)*.* Bulk G4 CUT&Tag derived from centrifugation-based protocol showed superior signal-to-noise ratio and sensitivity to weaker G4 signals (Additional file 1**: **Fig. S1A-D). To validate the single-cell G4 CUT&Tag workflow, we prepared a heterogeneous cell mix and attempted to resolve single cells into discrete groups recapitulating the original cell types based exclusively on G4 patterns. We mixed mouse embryonic stem cells (mESC) with mouse embryonic fibroblasts (MEF, NIH-3T3), and following BG4 antibody-based in situ labeling and tagmentation, we performed scCUT&Tag using the 10 × Genomics scATAC-seq kit. After processing the single-cell data we obtained 1,842 single cell profiles with 3,434 median reads passing quality filter. The median fraction of reads in peaks (FRIP) was 52%, indicating a high peak enrichment characteristic of CUT&Tag data [28, 29]. In order to determine whether standard clustering approaches are able to identify cell types based on the G4 signal, we performed dimensional reduction by latent semantic indexing (LSI) and uniform manifold approximation and projection (UMAP) followed by shared nearest neighbors (SNN) driven clustering by Seurat package. Using this approach with resolution parameter 0.1 we obtained two distinguishable clusters (Fig. 1B). Quality controls were on par with other high quality single-cell CUT&Tag datasets (Fig. 1C, Additional file 1: Fig. S2A, B). To annotate the two clusters, we first performed an overlap analysis applied on MACS2 peak sets coming from bulk G4 CUT&Tag experiments and validated that cluster 1 (mESC cluster hereafter) shows higher unique overlap (6,543/34,152 peaks) with G4 sites of mESC G4 than cluster 0 (1,257/31,019 peaks). Cluster 0 (MEF cluster hereafter) showed a higher unique overlap (3,321/31,019 peaks) with the MEF G4 peak set than cluster 1 (1,113/34,152 peaks) (Fig. 1D). Importantly, 88% of cluster 0 specific peaks, 93% of cluster 1 specific peaks, and 96% of shared peaks overlapped with at least one PQS (2.6 × enrichment in permutation test, z > 80, *p* < 0.001) (Fig. 1E, Additional file 1: Fig. S2C).

To further validate that the clustering into two populations was indeed driven by cell-type specific G4 patterns, we identified differential peaks (log2fc > 2) between cluster 0 and cluster 1 and calculated Pearson correlation with MEF and mESC bulk G4 CUT&Tag datasets. Cluster 0 strongly correlated (r = 0.74) with MEF bulk, whereas cluster 1 strongly correlated (r = 0.73) with mESC bulk, confirming that cell type was the predominant source of single cell variation in our experiment (Fig. 1F).

### Cluster-specific G4s reflect cell-type specific gene function and regulatory mechanisms

We wondered about the biological role of the cluster-specific peaks that reflected cell-type specific G4s and performed gene set enrichment analysis on the subset of promoter G4s. Interestingly, only 10%−28% of cluster-specific G4s intersected with ~ 30′000 RefSeq genes or 142,000 GENCODE transcript variant promoters, respectively, as opposed to 40–54% of the shared/constitutive G4s (Additional file 1: Fig. S2D, E). This suggested that cell-type specific G4s are more often formed at cis-regulatory elements than promoters, and more often at variants than canonical gene promoters. Genes with cluster-specific promoter G4s were enriched in GO terms relating to cell motility, migration, chemotaxis, cytoskeleton for cluster 0, signal transduction, developmental and morphogenesis terms for cluster 1 (Fig. 1G), directly reflecting known functional characteristics of the respective MEF and mESC cell types. Conversely, constitutive/shared promoter G4s were enriched in general GO terms, like DNA metabolism, protein modification, protein degradation, DNA damage and repair.

### Cluster-specific G4s correlate weakly with ATAC-seq chromatin accessibility

Next, we examined scG4 pseudobulk and bulk G4 signal around cluster-specific and shared peaks as heatmaps and average profile plots side-by-side with matching mESC and MEF bulk ATAC-seq (Fig. 1H). Superficially, ATAC-seq signal tracked well with G4 single-cell and bulk signals, in line with the common notion that G4 formation strongly correlates with chromatin accessibility. However, intersection of our scG4 peaks with 55,057 (MEF) or 62,739 (mESC) bulk ATAC-seq peaks showed that only 67% (cluster 0) and 48% (cluster 1) scG4 peaks overlapped with MEF and mESC ATAC-seq peaks, respectively (Additional file 1: Fig. S3A). Hence, a large number of scG4s did not overlap with strong chromatin accessibility. Moreover, direct comparison of scG4 pseudobulk versus matched MEF/mESC ATAC-signals showed that the signal of cluster-specific G4s correlated only weakly (Pearson r = 0.26–0.38) with chromatin accessibility (Fig. 1I), with many strong G4 peaks showing very low ATAC signal. Similarly, a fraction of constitutive/shared G4 peaks showed low accessibility in both mESC and MEF (Additional file 1: Fig. S3B). Importantly, this was not a function of noisy/stochastic scG4 signals as the pseudobulk scG4 signals from cluster 0 and cluster 1 correlated extremely well over constitutive/shared G4 peaks (Pearson r = 0.89, Additional file 1: Fig. S3B). Representative genome browser tracks show MEF and mESC-specific G4 peaks and an example of G4 peaks with low accessibility (Fig. 1J). Together, these results confirm that our single-cell G4 profiling via scCUT&Tag can detect G4 structures in individual cells with high specificity and has sufficient resolution to distinguish cell types based on their G4 landscape.

### The single-cell G4 landscape in postnatal mouse brain is distinct from accessibility

**Fig. 2:**
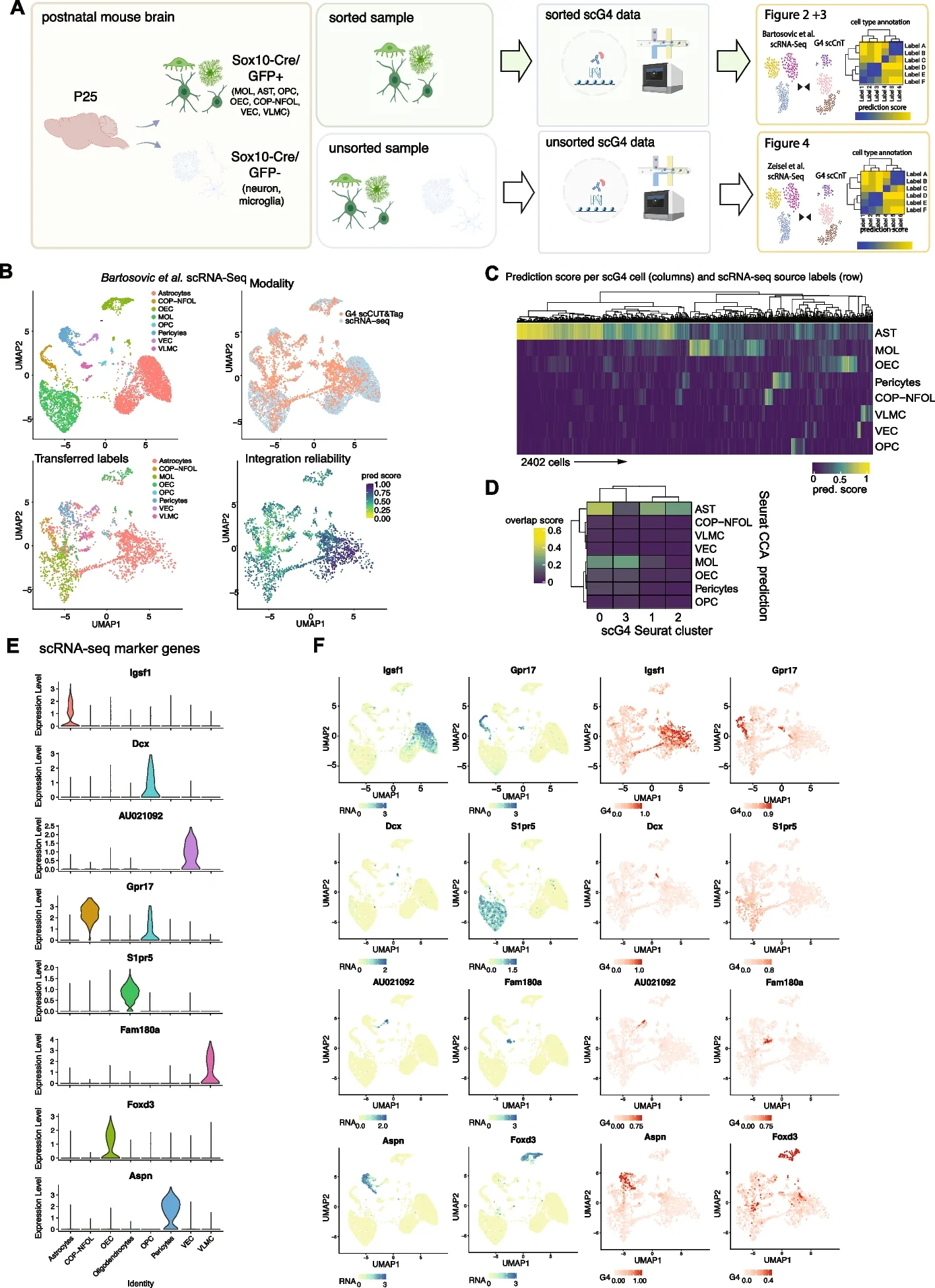
Integration of G4 scCUT&Tag data with scRNA-seq using Canonical Correlation Analysis (CCA) **A**. Schematic workflow for the study of single-cell G4 landscapes in postnatal mouse brain. **B**. Joint embedding of the GFP + scG4 cells and the scRNA-seq reference [30]. 5,488 scRNA-seq and 2,767 G4 cells (8,255 total). UMAPs show scRNA-seq ground truth (top left), modality (top right), transferred labels on scG4 cells (bottom left), and integration reliability of the same (bottom right). Abbreviations AST: astrocyte, MOL: mature oligodendrocyte, OPC: oligodendrocyte precursor, COP: committed oligodendrocyte precursor, OEC: olfactory ensheathing cells, NFOL: newly formed oligodendrocyte, VEC: vascular endothelial cells, VLMC: vascular and leptomeningeal cell. **C**. Heatmap of the class-transfer prediction scores for the GFP + G4 cells across the reference cell types. **D**. Cluster/prediction correspondence: Symmetric overlap-score heatmap quantifying the agreement between Seurat clusters and predicted cell types (fraction-of-cluster vs fraction-of-cell-type overlap). **E**. Output of the scRNA-seq marker analysis. Violin plots of the top differentially expressed marker gene per cell type, from SeuratFindAllMarkers. **F**. G4/RNA co-enrichment feature plots. For the same marker genes, UMAP feature plots on the shared embedding showing the G4 gene-activity score and the normalized scRNA-seq expression, demonstrating co-enrichment of G4 activity and expression within the corresponding cell types

Having established that scCUT&Tag allowed the deconvolution of cell-type-specific G4 structures in heterogeneous mixtures, we turned to the developing mouse brain, collecting P21 postnatal brain cells from transgenic mice expressing Sox10:Cre/Rosa26:(CAG-LSL-EGFP(RCE)) [30]. The reporter labels major brain cell types, including astrocytes (AST), mature oligodendrocytes (MOL), oligodendrocyte progenitor cells (OPC), committed oligodendrocyte precursors or newly formed oligodendrocytes (COP-NFOL), olfactory ensheathing cells (OEC), and vascular cells such as pericytes, vascular endothelial cells (VEC), and vascular and leptomeningeal cells (VLMCs). GFP- cells were more abundant in neurons and microglia relative to the GFP + population [30]. We conducted G4 scCUT&Tag experiments with GFP+ cell sorting (sorted) or without sorting (unsorted) and processed the GFP + sorted population using a standard Seurat workflow and QC (Fig. 2A, Additional file 1: Fig. S4A). Best clustering was achieved with a resolution of 0.8, yielding 4 clusters (Additional file 1: Fig. S4B). Peak calling yielded ~ 26,600 peaks in total, of which 84–87% per cluster overlapped with at least one PQS (2.8 × enriched in permutation test, z > 65, *p* < 0.001) (Additional file 1: Fig. S4C). Despite a significant overlap (77%) of scG4 peaks with scATAC peaks (Additional file 1: Fig. S4D), correlation of scG4 and scATAC signal was very weak (Pearson r < 0.3) (Additional file 1: Fig. S4E). All of these observations support the notion that, while G4 structures often associate with larger regions of open chromatin, the extent of G4 formation is determined by additional and/or independent mechanisms.

### Annotation of G4 scCUT&Tag clusters using co-embedding and label transfer

To annotate cell types, we used a labeled single-cell RNA-Seq (scRNA-seq) dataset (Additional file 1: Fig. S4F) from postnatal mouse brain of the same age [30] and performed horizontal data integration between the single-cell G4 and transcriptomics modalities by co-embedding the datasets using mutual nearest neighbors and canonical correlation analysis (CCA, implemented in Seurat) (Fig. 2B). To anchor features between the datasets, we considered G4 scCUT&Tag signal over the gene body and promoter region in the single-cell G4 modality. We computed gene activity scores using the GeneActivity function (Signac package) and set these scores as the default assay for the G4 analysis. Following anchor identification across the datasets (FindTransferAnchors function, Seurat), we performed data imputation (TransferData function, Seurat) with three optimized settings: (1) we prioritized the top variable genes (*n* = 2,000) from the mean variability plot of the scRNA-seq dataset; (2) we used the previously identified anchors between the two objects as our anchor set; and (3) we applied LSI as the dimensionality reduction technique for anchor building. The resulting embedding resulted in reasonable prediction scores for all scRNA-seq populations (Fig. 2B, Additional file 1: Fig. S4G). Comparing prediction scores for each cell and source label, we could confirm that most cells received high prediction scores only for a single source label, allowing them to be confidently assigned to a single cell type (Fig. 2C). AST labels showed the largest number of assignments with very high (> 0.8) scores. A minority of cells shows similar prediction scores for multiple labels, e.g. AST + MOL (Fig. 2C). We then assessed cluster similarity across the two modalities and calculated overlap scores (Fig. 2D, detailed in Materials and Method and in [34]). We observed that cluster 2 of the sorted dataset consists of only ASTs, while cluster 0 and cluster 1 contain multiple cell types (mainly ASTs and MOLs). Finally, using the gene expression markers defined previously from the scRNA-seq dataset (Fig. 2E) and the co-embedded G4 data, we asked whether gene expression and G4s show any co-enrichments at the level of cell types. The feature plots showed (Fig. 2F) that G4 signals at the marker genes correlated with their scRNA-seq level in closely co-embedded cells. Together, we found that the GFP + population could be deconvoluted into major cell types, demonstrating cell-type-specific G4 heterogeneity, but the overall separation power of G4 signal is weaker than scRNA-seq and scATAC-seq. Successful label transfer relied on the fact that G4 signal at the marker genes increased with their expression, in line with the commonly observed positive correlation of G4s and active transcription [7, 15].

### Integration through reliability modeling enhances the annotation of G4 scCUT&Tag clusters

**Fig. 3:**
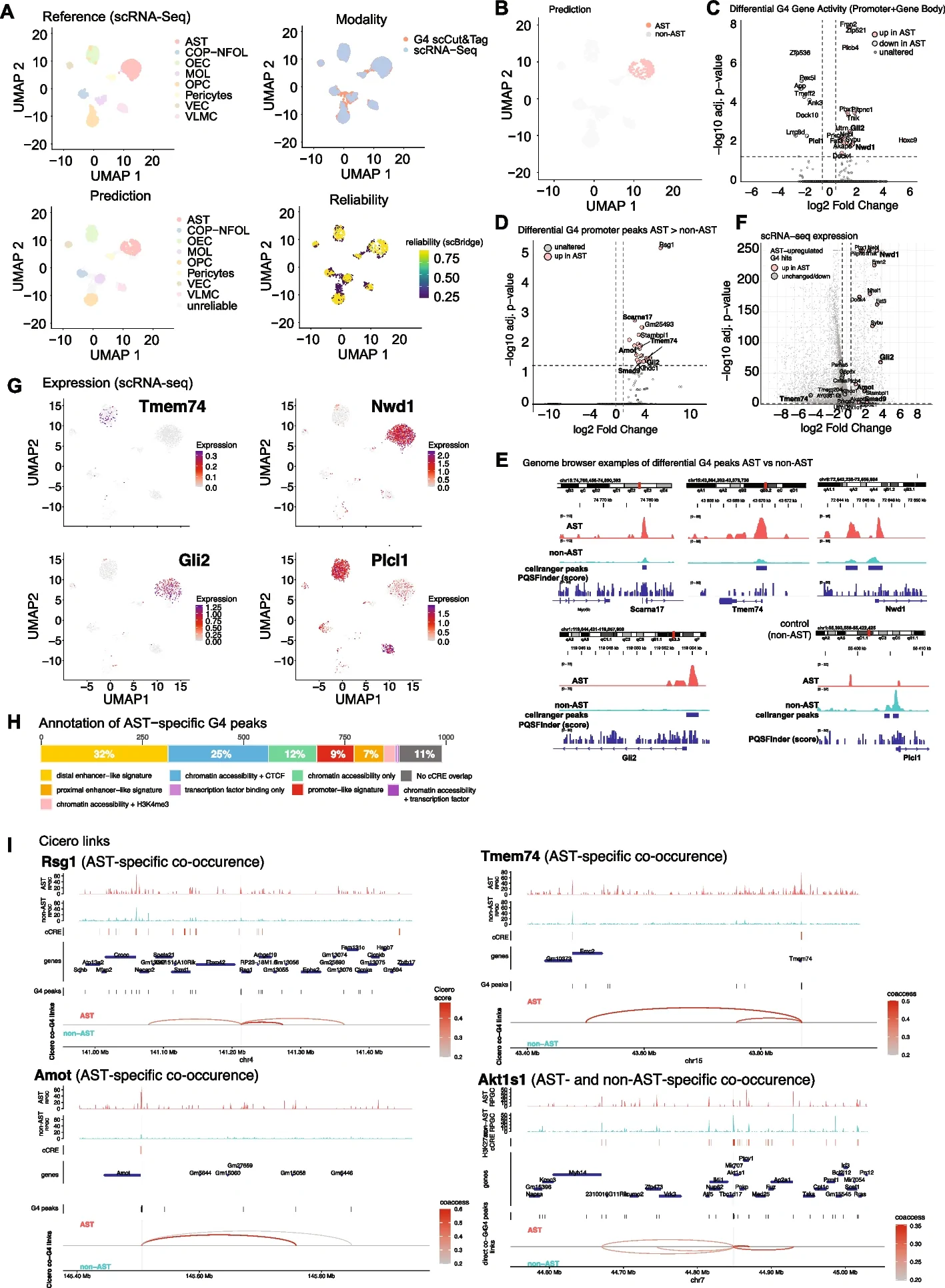
Integration through reliability modeling enhances the annotation of G4 scCUT&Tag clusters **A**. scBridge joint-embedding UMAP. Four views share a co-embedding of 5,488 scRNA-seq and 2,767 scG4 cells (8,255 total). Cells are colored by cell types, predicted labels, integration reliability and modality, respectively. **B**. UMAP indicating predicted scG4 AST population **C**. Gene-level analysis of astrocyte (AST) vs non-AST differential G4s. Volcano plot shows the differential G4 activity scores. Dashed lines denote the thresholds of the differential analysis: average |log_2_ fold change| > 0.5 & adj. *p*-value < 0.05. **D**. AST-specific promoter G4s. Volcano plot shows the differential G4 signal at promoters. Dashed lines denote the thresholds of the differential analysis: average |log_2_ fold change| > 0.5 & adj. *p*-value < 0.05. **E**. scG4 pseudobulk coverage tracks (RPGC normalized) show examples of genes (highlighted in bold on the volcano plot) with significantly higher promoter G4 signals in AST cells. PQS (pqsfinder score 20) are shown. **F**. Differential scRNA-seq gene expression analysis of astrocyte (AST) vs non-AST cells used for scBridge label transfer [30]. Volcano plot shows genes with AST-specific G4s. Dashed lines denote the thresholds of the differential analysis: average |log_2_ fold change| > 0.5 & adj. *p*-value < 0.05. **G.**) UMAP featureplots showing scRNA-seq signal [30] of selected genes with AST-specific (*Tmem74*, *Nwd1*, *Gli2*) and non-AST (*Plcl1*) promoter G4s. **H**. Functional annotation of AST-specific G4s. 994 AST-specific G4 peaks annotated with ENCODE4 cCRE classes. **I**. Cicero reveals cell-type specific G4 networks. Co-incidence analysis of G4 signals in single cells identifies examples of strong links between promoters and distal G4 sites in a cell-type-specific manner. Coverage plots show normalized G4 signal in AST (red) and non-AST (cyan) subpopulations. scG4 peaks and cCREs[36] are indicated. Cicero links (score > 0.2) are shown for AST and non-AST populations

To attempt a more adaptive cell type annotation for the GFP + brain sample, we used scBridge [35] which is a semi-supervised method performing the integration using heterogeneous transfer learning. scBridge performs annotation through a progressive learning approach, beginning with cells in the scCUT&Tag data that have the most similar profiles to scRNA-seq cells based on higher positive correlations with gene expression. This technique allowed us to leverage not only the cell heterogeneity of the sample but also the similar enrichment patterns observed in the co-embedded data space. To illustrate the scBridge driven integration of scRNA-seq and G4 scCUT&Tag data, we visualized the process with UMAPs, highlighting reference cell type labels from the reference scRNA-seq, data modalities, predicted labels on the G4 scCUT&Tag layer or reliability score (Fig. 3A).

As demonstrated for scBridge [35], it was discriminative, assigning high reliability to a substantial portion of cells. 2,083 cells from 2,767 had reliability score > 0.9 (Fig. 3A), at the expense of assigning more cells to an ‘unreliable’ assignment. To select a specific cell type for further characterization, we visualized Seurat prediction scores and predicted labels on the scBridge UMAPs (Additional file 1: Fig. S5A) and calculated overlap scores across Seurat clusters and scBridge predictions (Additional file 1: Fig. S5B), as well as between reliable Seurat predictions (prediction score cutoff: 0.5) and scBridge predictions (Additional file 1: Fig. S5C). Because prediction scores for the AST population were most robust between the different label transfer methods, we further investigated the G4 landscape of this population.

### Characterizing the astrocyte-specific G4 landscape

While label transfer methods operate on the premise that marker features are co-regulated, and here we relied on an expected correlation of transcriptional activity and G4 formation to transfer labels from a matched scRNA-seq dataset, we sought to investigate in a more unbiased manner the nature of cell-type specific G4s. First, we sought to identify AST-specific G4s by calling differential G4 features between scBridge-predicted astrocytes (AST, *n* = 183) and non-astrocytes (non-AST, excluding "unreliable" labels, *n* = 1,920). We performed Seurat FindMarker using the ‘Gene Activity’ (Fig. 3B,C), which represents the aggregated G4 signal across the entire gene (total 17,210 genes). Since G4s within the gene body could represent enhancers and other regulatory elements unrelated to the gene, we also sought to more specifically query for promoter G4s. To this end, we quantified differential G4 signals at 8,833 promoters that featured a G4 peak within ± 2 kb of the TSS and performed an analogous differential analysis to identify genes with AST-specific promoter G4s (Fig. 3D). We identified 18 genes with AST-specific G4 enrichment using FindMarker, and 23 genes with an AST-specific promoter G4 (|lfc| > 0.5, FDR < 5%). Only one gene (*Gli2*) was shared between those sets, indicating that promoter and gene-average signals reported on different underlying mechanisms. Example genome browser tracks show AST-specific promoter G4s for the genes *Scarna17*, *Tmem74*, *Nwd1*, *Gli2*, and a non-AST signal for *Plcl1* (Fig. 3E), confirming the results of the differential analysis. We next wondered if AST-specific promoter G4s correlated with AST-specific gene expression (irrespective if a cause or a consequence of the gene activity). To this end, we calculated differential expression between AST-labeled and non-AST scRNA-seq cells that provided the ground truth for the label transfer (Fig. 3F). Of the 23 genes with AST-specific promoter peaks, only 4 were significantly upregulated by scRNA-seq, 5 were downregulated, and 3 not significantly changed (|lfc| > 0.5, FDR < 5%, the remaining did not exist in the scRNA-seq assay). Inspection of the feature map confirmed that the AST-specific G4 promoter signal (Fig. 3E) coincided with AST-specific expression of the *Nwd1* and *Gli2* genes. Our non-AST-specific G4 control gene, *Plcl1*, was indeed predominantly expressed in other (OEC and VLMC) cell types. In contrast, *Tmem74*, which showed a strong promoter G4 in AST (Fig. 3E), did not show measurable expression in AST, instead appeared to be expressed in OEC (Fig. 3G). This suggested that differential G4 structures between cell types were not merely a function of gene activity, but may reflect independent changes in the local chromatin structure and organization. In order to expand on this concept, we analyzed the remaining AST-specific G4 peaks that did not overlap with annotated promoters in Fig. 3D. There were 994 AST-specific G4 peaks called in total (|lfc| > 0.5, FDR < 5%), which we intersected with ENCODE4 cCREs (Fig. 3H). Interestingly, 32% of AST-specific G4 overlapped with distal enhancer-like signatures, whereas 25% were associated with CTCF binding. Overlap of G4s and CTCF binding sites has been previously noted and a potential cooperation has been proposed [28, 37]. Together with additional promoter- and cis-regulatory element categories, a total of 89% of AST-specific G4 structures fell into annotated regulatory regions.

Single-cell epigenomics data provides a unique opportunity to identify interactions between regulatory elements through co-incidence detection in single-cells [38]. To explore this, we applied Cicero [38] on the normalized single-cell G4 signals from the annotated GFP + scCUT&Tag dataset. Originally developed for single-cell chromatin accessibility data, Cicero is an unsupervised method that predicts cell-type-specific interactions between cis-regulatory regions and works by measuring the correlation between pairs of proximal links. Following LSI preprocessing and UMAP reduction, Cicero identified 22,928 and 2,758 correlated features with co-accessibility scores above 0.2 in AST and non-AST cells, respectively. Representative examples of regions with AST-specific promoter G4 anchors (*Rsg1*, *Tmem74*, *Amot* genes) and high co-accessibility scores (> 0.2) demonstrated physical interactions between proximal and distal G4 sites overlapping with H3K27-acetylated putative cis-regulatory elements [36] (Fig. 3I). Importantly, the loops formed predominantly between AST-specific G4 peaks even if they were interspersed with constitutive G4s in the same genomic region, raising the interesting hypothesis that G4s may coincide with or contribute to cell-type specific 3D genome organization principles. We also found examples where constitutive promoter G4s formed loops with distinct G4s present in only the AST- or non-AST populations (*Akt1s1* and *Sox10*, Fig. 3I, Additional file 1: Fig. S5D).

Altogether, G4 scCUT&Tag facilitates the detection of interacting G4 sites that colocalize with cis-regulatory regions and single-cell level examination of G4 interactions.

### Integration of the GFP- cluster from the unsorted G4 scCUT&Tag dataset with neuronal scRNA-seq

**Fig. 4:**
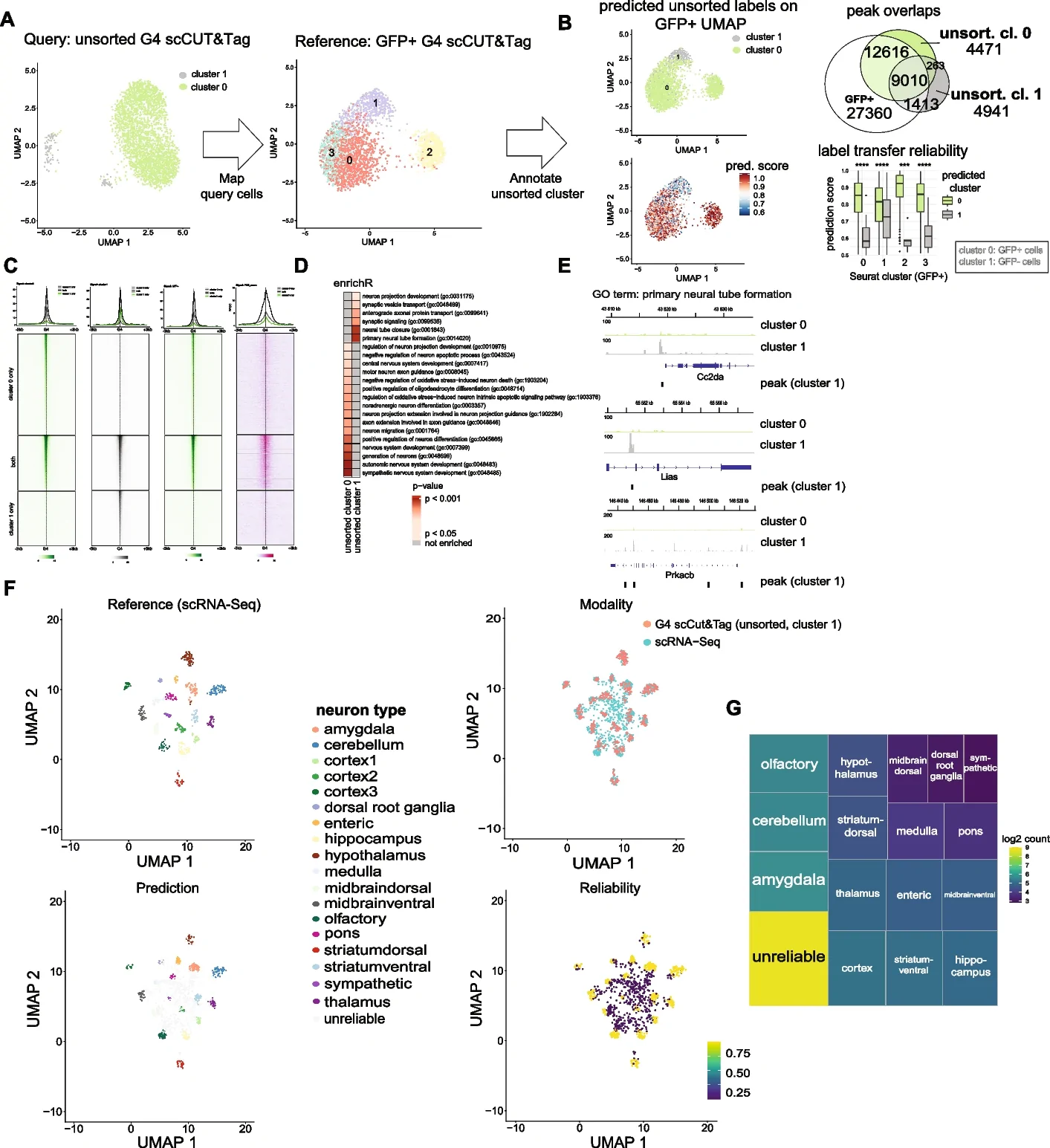
Integration of the GFP- cluster from the unsorted G4 scCUT&Tag dataset with neuronal scRNA-seq aids the identification of neuronal cell types. **A**. Projection of the unsorted scG4 CUT&Tag data (containing GFP- and GFP + fractions) as a query onto the GFP + dataset as a reference. **B**. UMAPs show the mapped query labels and the prediction scores upon the label transfer. Venn diagram indicates the peak overlaps between the GFP + dataset and unsorted clusters. Boxplot shows the distribution of the prediction scores between the unsorted clusters at the level of GFP + Seurat clusters. **C**. Heatmaps of G4 occupancy at unique and overlapping peak regions. Signals are normalized by RPGC. Additionally, pqsfinder scores are shown **D**. enrichR gene ontology analysis on the cluster-specific peak sets. Heatmap indicates the CNS-related GO terms and the corresponding *p*-values. **E**. Genome viewer tracks display representative cluster 1-specific G4 peaks related to the GO term primary neural tube formation (GO:0014020). **F**. UMAP visualizations of the joint embedding of *Zeisel *et al*.* scRNA-seq data and the cluster 1 of the unsorted dataset. Cells are colored according to neuron types, data modality, predicted labels, and integration reliability, respectively. **G**. Treemap quantifying the predicted neuron types. The color scale represents the log_2_-transformed count of each neuron type inferred from label imputation

To further explore the G4 landscape of the developing brain, we performed an additional G4 scCUT&Tag experiment on the unsorted mouse brain tissue. Overall data quality was consistent with prior experiments and > 86% of called peaks overlapped with G4-forming motifs (Additional file 1: Fig. S6). Initial dimensionality reduction of the unsorted single cells (*n* = 3,910) and clustering with a low-resolution parameter (set to 0.1) resulted in one major cluster 0 and a smaller cluster 1. We mapped the unsorted cells (query) to the GFP + cells (reference) and assigned labels accordingly (Fig. 4A). Cells from cluster 0 showed significantly higher prediction scores (mean: 0.84) than those from cluster 1 (mean: 0.69) (Fig. 4B), suggesting that cluster 0 contained most of the GFP + population. To further characterize these clusters, we performed peak calling and identified 4,941 peaks unique to cluster 1 of the unsorted population, which were absent in both cluster 0 and the GFP + population (Fig. 4B). Pseudo-bulk showed a strong signal for cluster 1-specific peaks that was indeed absent in cluster 0 and the GFP + cells. These cluster 1-specific peaks were enriched in genes associated with GO terms such as neural tube closure and primary neural tube formation, suggesting that cluster 1 consists of neuronal cell types (Fig. 4D). For instance, genes such as *Cc2d1a*, *Lias*, and *Prkacb*, which are involved in primary neural tube formation, showed unique G4 signals in cluster 1 (Fig. 4E) whereas mature oligodendrocyte marker *Klk6* showed a strong cluster 0-specific G4 (Additional file 1: Fig. S7A). To further dissect the cell types of cluster 1, we attempted the co-embedding of our unsorted cluster 1 cell population with scRNA-seq from an adolescent mouse brain. For the data integration, we used neuron scRNA-seq data from the mouse brain atlas of *Zeisel *et al*.* [33] as ground truth considering 13 distinct tissues. Indeed, the resulting cell embeddings (Fig. 4F) displayed clear patterns corresponding to neuronal subtypes, enabling confident annotation (scBridge reliability score > 0.90) of 444 out of 963 cluster 1 cells. These annotated cells spanned various neuronal types, including those from the amygdala, thalamus, cortex, striatum, and cerebellum (Fig. 4G), but also approximately 50% of the cells remained unannotated. This might be due to the limitations of the label transfer or the presence of cell types not represented in the scRNA-seq reference, such as non-neuronal populations.

## Discussion

Here, we demonstrate that G4 scCUT&Tag enables profiling of endogenous G-quadruplex landscapes at single-cell resolution in heterogeneous primary tissue. In a defined mixture of mESCs and MEFs, G4 profiles distinguished the two cell populations and recapitulated their corresponding bulk G4 CUT&Tag landscapes. Application to the postnatal mouse brain further revealed G4 heterogeneity across neural cell types. Integration with scRNA-seq using CCA and scBridge enabled annotation of major glial and neuronal populations, demonstrating that cell-type composition is reflected at the level of G4 formation. Together, these results extend G4 scCUT&Tag from cultured cell lines to complex primary tissue and demonstrate its utility for investigating cell-type-specific G4 landscapes [32].

An important observation was that G4 landscapes were related to, but distinct from, chromatin accessibility. Although G4 structures frequently occurred within accessible chromatin, G4 and ATAC-seq peaks showed only partial overlap, and G4 signal intensity correlated only weakly with chromatin accessibility. Thus, while G4 structures are often associated with open chromatin, the extent of G4 formation appears largely uncoupled from accessibility. Integration with scRNA-seq similarly revealed an overall positive relationship between G4 enrichment and transcription, consistent with previous studies associating promoter G4s with transcriptionally active genes [7, 13, 39–41]. However, only a subset of genes with astrocyte-specific promoter G4s showed corresponding differential expression, suggesting that cell-type-specific G4 formation is not merely a consequence of gene activity.

Cell-type-specific G4s were also frequently associated with putative cis-regulatory elements. In astrocytes, 89% of differential G4 structures occurred within annotated regulatory regions, including distal enhancer-like signatures and CTCF-associated sites. Analysis of single-cell G4 signals with Cicero further identified correlated proximal and distal G4 sites, including examples overlapping H3K27ac-marked putative cis-regulatory elements. These observations are consistent with previous associations between G4s and regulatory chromatin and raise the possibility that G4s coincide with cell-type-specific 3D genome organization.

A current limitation is that G4 scCUT&Tag provides weaker unsupervised separation of closely related cell populations than scRNA-seq or scATAC-seq, and annotation of heterogeneous brain populations therefore benefited from integration with reference transcriptomic datasets. Increasing the number of unique fragments per cell may further improve the resolution of G4 profiles. In addition, paired multi-omics approaches that simultaneously measure G4 and gene expression in the same cell could provide deeper insight into their relationship. Overall, G4 scCUT&Tag provides a framework for examining cell-type-specific G4 landscapes in complex tissues and their relationships with transcription and chromatin organization.

## Conclusions

In conclusion, we demonstrate that G4 scCUT&Tag enables single-cell profiling of endogenous G4 landscapes in primary, heterogeneous tissue. Applied to the postnatal mouse brain, the method revealed cell-type-associated G4 heterogeneity and identified distinct G4 signatures across glial and neuronal populations. Integration with single-cell transcriptomic data further enabled cell-type annotation and revealed an association between cell-type-specific G4 enrichment and transcriptional activity. Moreover, the identification of G4 sites associated with putative cis-regulatory elements and predicted long-range interactions extends the utility of G4 scCUT&Tag beyond local G4 mapping. Together, these findings establish G4 scCUT&Tag as a framework for investigating the cellular heterogeneity of G4 landscapes in complex tissues and provide a basis for exploring their relationships with gene regulation and other layers of chromatin organization.

## Methods

### Bulk G-quadruplex CUT&Tag with centrifugation

Bulk G-quadruplex CUT&Tag experiments were performed as in Lyu et al. [28], but without Concanavaline A beads to better maintain the integrity of the nuclei. All the centrifugation steps in the protocol were performed at 600 g or 1700 rpm at 4℃ with swing bucket centrifuge. 200′000 cells were harvested by centrifugation, resuspended in wash buffer (20 mM HEPES pH 7.5, 150 mM NaCl, 0.5 mM spermidine) and pelleted again, incubated in 200 μl of antibody binding buffer (wash buffer with 2 mM EDTA, 0.01% NP-40, 0.05% digitonin and 1% BSA) containing 2 μg of BG4 antibody (Addgene #55756) with rotation overnight at 4℃. The next day, cells were washed with 200 μl of dig-wash buffer (wash buffer with 0.01% NP-40, 0.05% digitonin and 1% BSA) three times by centrifugation at 600 g or 1700 rpm for 4 min with swing bucket centrifuge. After washing, cells were incubated with 1:100 dilution of anti-FLAG antibody (Sigma, F1804) for one hour at room temperature with rotation. Cells were washed with 200 μl of dig-wash buffer to remove unbound anti-FLAG antibodies. Anti-FLAG treated cells were incubated with 1:100 dilution of anti-mouse antibody (Sigma, M7023) in a dig-wash buffer for one hour at room temperature, followed by washing with 200 μl of dig-wash buffer three times. After antibody incubation, cells were incubated with 1:200 dilution of assembled pA-Tn5 in 200 μl of dig-300 buffer (20 mM HEPES pH 7.5, 300 mM NaCl, 0.5 mM spermidine, 0.01% NP-40, 0.05% digitonin and 1% BSA) for 1 h at room temperature. After pA-Tn5 binding, cells were washed with 200 μl of dig-300 buffer three times. Afterwards, pA-Tn5 bound cells were incubated in 200 μl of tagmentation buffer (dig-300 buffer with 10 mM MgCl_2_) at 37℃ for 1 h without shaking. After tagmentation, genomic DNA was extracted and purified with DNA Clean & Concentrator-5 (Zymo research, D4013). To generate the G4 library, purified genomic DNA was amplified with the universal i5 primer and barcoded i7 primer using NEBNext Ultra II Q5 Master Mix (NEB, M0544). The library PCR products were cleaned up with Agencourt AMPure XP beads (Beckman Coulter, A63881) and sequenced on an Illumina Nextseq 2000 instrument.

### pA-Tn5 loading

Mosaic-end adapter A (Tn5ME-A, TCGTCGGCAGCGTCAGATGTGTATAAGAGACAG) and Mosaic-end adapter B (Tn5ME-B, GTCTCGTGGGCTCGGAGATGTGTATAAGAGACAG) oligonucleotides were each annealed with Mosaic-end reverse oligonucleotides (Tn5MErev, 5′-(phos)CTGTCTCTTATACACATCT-3′). To anneal, the oligonucleotides were diluted to 200 µM. Each pair of oligos, Tn5MErev/Tn5ME-A and Tn5MErev/Tn5ME-B, was mixed and annealed separately. The loading of pA–Tn5 with the oligonucleotides was performed by incubating 20 μl of pA-Tn5 (5.5 μM) with 2.2 μl of annealed Tn5MErev/Tn5ME-A (100 μM) and 2.2 μl of annealed Tn5MErev/Tn5ME-B (100 μM) at room temperature for 1 h.

### Cell line culture

Mouse embryonic stem cells (RW.4 129X1/SvJ, authenticated by and acquired from Karolinska Institutet Gene Targeting Facility) were cultured feeder-free in 0.1% gelatin-coated flasks (Sigma, G1890) under standard conditions (5% CO_2_, 90% humidity, 37 °C) in KnockOut DMEM (LifeTechnologies, 10,829,018), 2 mM alanyl-glutamine (Sigma, G8541), 0.1 mM non-essential amino acids (Sigma, M7145), 15% fetal bovine serum (FBS) (Sigma, F7524), 0.1 mM β-mercaptoethanol (Sigma, M3148), ESGRO Leukemia Inhibitory Factor (LIF) (Millipore, ESG1107), 1 μM PD0325901 (PZ0162-25MG) and 3 μM CHIR99021 (SML1046-25MG). NIH-3T3 (ATCC) cells were cultured in DMEM (Gibco, 31,966,047) supplemented with 10% fetal bovine serum (FBS; Gibco, 26,140,079) and 1 × penicillin/streptomycin (Sigma, P4333). Cell lines were routinely tested for mycoplasma contamination.

### Animals

The mouse line used in this study was generated by crossing Sox10:Cre animals7 (The Jackson Laboratory mouse strain 025807) on a C57BL/6 j genetic background with RCE:loxP (EGFP) animals (The Jackson Laboratory mouse strain 32,037-JAX) on a C57BL/6xCD1 mixed genetic background. Females with a hemizygous Cre allele were mated with males lacking the Cre allele, while the reporter allele was kept in hemizygosity or homozygosity in both females and males. In the resulting Sox10:Cre-RCE:LoxP (EGFP) animals the entire oligodendrocyte lineage was labeled with EGFP [30]. All animals were free from mouse viral pathogens, ectoparasites and endoparasites, and mouse bacteria pathogens. Mice were kept with the following light/dark cycle: dawn 6:00–7:00, daylight 7:00–18:00, dusk 18:00–19:00, night 19:00–6:00 and housed to a maximum number of five per cage in individually ventilated cages (IVC sealsafe GM500, tecniplast). General housing parameters such as relative humidity, temperature, and ventilation follow the European convention for the protection of vertebrate animals used for experimental and other scientific purposes treaty ETS 123, Strasbourg 18.03.1986/01.01.1991. In brief, consistent relative air humidity of 55 ± 10%, 22 °C and the air quality is controlled with the use of standalone air-handling units supplemented with HEPA-filtrated air. Monitoring of husbandry parameters is done using ScanClime (Scanbur) units. Cages contained hardwood bedding (TAPVEI, Estonia), nesting material, shredded paper, gnawing sticks, and card box shelter (Scanbur). The mice received regular chow diet (either R70 diet or R34, Lantmännen Lantbruk). Water was provided by using a water bottle, which was changed weekly. Cages were changed every other week. All cage changes were done in a laminar air-flow cabinet. Facility personnel wore dedicated scrubs, socks, and shoes. Respiratory masks were used when working outside of the laminar air-flow cabinet. Animals were sacrificed at juvenile stages (P21). The mice received a sublethal dose of anesthetics, then assessed if they reached a surgical plane of anesthesia by pinching the toe. When the reflexes were absent (no response to stimulation), a perfusion with commercial 1X PBS pH 7.4 was performed. All experimental procedures on animals were performed following the European directive 2010/63/EU, local Swedish directive L150/SJVFS/2019:9, Saknr L150 and Karolinska Institutet complementary guidelines for procurement and use of laboratory animals, Dnr. 1937/03–640. The procedures described were approved by the local committee for ethical experiments on laboratory animals in Sweden (Stockholms Norra Djurförsöksetiska nämnd), license number 144/16, 1995_2019 and 7029/2020. In accordance with the ARRIVE Guidelines Essential 10, Points 8. Animals, 9. Experimental procedures and 10. Results are reported above. Points 1.−7. (study design, sample size, inclusion and exclusion criteria, randomization, blinding, outcome measures, statistical methods) were not deemed relevant since no comparative study was performed.

### Tissue dissociation

Mice were sacrificed, perfused with 1 × PBS, pH 7.4 and the brain was removed. The brain was dissociated into single-cell suspension using Neural Tissue Dissociation Kit P (Miltenyi Biotec, 130–092–628) according to the manufacturer’s protocol. For mice at P21 stage, myelin was removed using debris removal solution (Miltenyi Biotec, 130–109–398) according to the manufacturer’s instructions. Single-cell suspension was filtered through a 50 µm cell strainer and briefly stained with 1:5000 diluted DAPI (1 mg ml − 1) to assess cell viability. For FACS, cells were resuspended in 1 × PBS supplemented with 1% BSA and 2 mM EDTA and GFP + cells were sorted using fluorescent-assisted cell sorting (FACS Aria III or FACS Aria Fusion) directly into 500 μl of antibody buffer.

### Single cell G-quadruplex CUT&Tag

Single cell workflow with 10 × platform was performed as in Bartosovic et al. [30]. For mESC-MEF, 100′000 mESC and 100′000 NIH-3T3 cells were mixed before G4 antibody incubation. All incubation and washes are the same as in the case of bulk G4 CUT&Tag. After tagmentation, the sample was centrifuged for 3 min at 300 g and resuspended in 15–25 μl of 1 × PBS + 1% BSA and processed using 10 × Chromium Single Cell ATAC-Seq kit. Final library amplification was performed according to the Chromium Single Cell ATAC Library kit manual sequenced on an Illumina Nextseq 2000 instrument.

### Bulk CUT&Tag analysis

Bulk CUT&Tag fastq files were processed through the nf-core/atacseq pipeline (v1.2.1). Essentially, reads were aligned to the mm10 reference genome with BWA (v0.7.17-r1188), deduplicated with Picard (v2.23.1), peaks were called with MACS2 (v2.2.7.1). deepTools (v3.5.2) [42] bamCoverage was used to produce bigwig files using RPGC (1 × Genome coverage) normalization.

### scCUT&Tag data processing and explorative analysis

#### Fastq alignment

One library of the mESC-MEF sample and one library for the sorted (GFP + cells) or unsorted (GFP- and GFP + cells) each postnatal mouse brain cells of Sox10:Cre/Rosa26:(CAG-LSL-EGFP) animals on P21 were prepared and sequenced on a Nextseq 2000 instrument. Fastq files were initially processed using the count function of 10X Genomics Cell Ranger ATAC software package (cellranger-atac-2.0.0, https://github.com/10XGenomics/cellranger-atac) with standard parameters. Reads were aligned to the mm10 genome reference. Cell Ranger ATAC identified 3,434, 1,983 and 1,511 median high-quality fragments per cell in the mESC-MEF, sorted and unsorted mouse brain samples, respectively.

#### Quality control

Processing of Cell Ranger ATAC H5 files and the subsequent single-cell analysis workflow were done in R using packages Seurat (v5.0.3) and Signac (v1.13) [43]. Chromatin assay object was created from features detected at least in 10 cells and from cells including at least 200 features. Doublets (Additional file 1: Fig. S1E) were eliminated by the R package called DoubletFinder [44] (https://github.com/chris-mcginnis-ucsf/DoubletFinder) with parameters pN = 0.25, pK = 0.09, nExp = 0.04, principal components 1:10. Quality measure plots, such as the one in Fig. 1C, were visually inspected at the level of Seurat clusters and used for quality assurance. FRiP of the bulk CUT&Tag experiments was calculated with Subread (v2.0.3) featureCounts [45].

#### Dimensionality reduction and clustering

After quality control and filtering, the read count matrix was transformed by term frequency inverse document frequency (TF-IDF) transformation followed by partial singular value decomposition. UMAP [46] preceded by latent semantic indexing (LSI) was run on the 2:30 dimensions, omitting the first component that highly correlates with sequencing depth. For the optimization of UMAP parameters we tested the number of neighbors (step between 5 and 50 by 2) and minimum distance (tweaked 25 values between 0.001 and 0.5). All cluster definition was performed by FindClusters of Seurat, so that the algorithm was set to smart local moving (SLM) [47] and the optimal resolution and granularity of clustering was tuned by the resolution parameter (step between 0.1 and 1 by 0.1). Eventually, for identification of cell origin in the mESC-MEF and in the unsorted mouse samples, our final parameter set was next: resolution 0.1, minimum distance 0.4, number of neighbors 30, SLM algorithm. For the sorted mouse sample, the parameter set remained unchanged, except for the resolution, which was adjusted to 0.8.

#### Peak calling and bam computation

Cluster-specific peaks were retrieved by Signac’s CallPeaks function using a simple MACS2 [48] peak calling (with the default parameters of CallPeaks) and with group.by = “seurat_clusters” to call peaks on each cluster separately. Generating bam files from each Seurat cluster was performed by the filterbarcodes function of the package sinto (https://timoast.github.io/sinto/). The retrieved bam files were converted to RPGC normalized bigwig files by bamCoverage of deepTools.

#### Finding marker regions

The term marker is used to refer to a significant differentially G-quadruplex region between any cluster to all other clusters. Marker regions for each cluster were calculated using the FindAllMarkers function from the Seurat package. Marker detection was performed using the logistic regression model of the function, as suggested by [49], and added the total number of fragments as a latent variable to mitigate the effect of differential sequencing depth on the result. In the comparison of AST and non-AST cells (Fig. 3) the gene activity matrix (generated by the Signac’s GeneActivity function that aggregates the counts over the gene body and promoter region) was set as input, so that the gene activity scores were generated by default base extension arguments (extend.upstream = 2000, extend.downstream = 0).

*Cicero*. Interplays across G4 sites were analyzed by the R package called Cicero [38]. G4 peaks were set as input with UMAP reduction and LSI preprocessing step. For finding cis-G4 networks, the “cutoff override” parameter was set to 0.2. Predictions were quantified by the co-accessibility score showing how correlated two G4 sites are in the cells. Visualization of the sites was made by the plot_connections function of the package and IGV.

#### Putative G4 sequence score (PQS score)

PQS scores, used to quantify the G-quadruplex-forming potential of the peak regions, were calculated using the pqsfinder R package [50].

#### Enrichment analysis

Enrichment analysis for gene ontology was done by the R package enrichR (v3.2) [51] using the “GO_Biological_Process_2023” database. For visualization, we retained only the GO terms connected to the CNS.

#### Visualizations

IGV (Integrative Genomics Viewer) [52] tool was used for the visualization of bigwig tracks and bed files. Profile plots, heatmaps and fingerprint plot were generated by deepTools. All further R visualization was done by ggplot2. [53]

### scRNA-seq data processing

*Bartosovic *et al*. scRNA-seq.* scRNA-seq reads derived from GFP + cells were aligned to the mm10 reference genome using Cell Ranger. The resulting read count matrix was processed with Seurat, including log normalization to 10,000 counts (using the NormalizeData function), identification of variable features (FindVariableFeatures function with selection.method = "vst"), scaling (ScaleData function), and dimensionality reduction via PCA (RunPCA function) and UMAP (RunUMAP function). Gene expression markers were calculated by the FindAllMarkers function with min.pct = 0.5.

*Zeisel *et al*. scRNA-seq.* Loom files corresponding to neurons from adolescent mouse brains were downloaded in Level 2 format from the Sten Linnarsson Mouse Brain Atlas [33] (see Data Availability). These files were further handled by the loomR package (https://github.com/mojaveazure/loomR). Afterwards, the neuronal data were processed using the same methods applied to the *Bartosovic *et al*.* scRNA-seq dataset.

### scCUT&Tag integration with scRNA-seq data

#### Integration using CCA of Seurat

Identification of anchor correspondences between the G4 scCUT&Tag of sorted mouse brain sample and *Bartosovic *et al*.* scRNA-seq dataset (both derived from Sox10:Cre/Rosa26:(CAG-LSL-EGFP) mice on P21) was identified using the FindTransferAnchors function of Seurat using our scCUT&Tag data as query and the labeled scRNA-seq data as reference. In the case of scCUT&Tag query the gene activity score assay was used for the integration according to the G4 enrichment of gene promoter and gene body as previously described [43]. The computed anchor points represent two cells (with one cell from each dataset) that originate from a common biological state. Prior to anchor detection the datasets were placed in a shared low-dimensional space by CCA [54]. Following the dimensional reduction nearest neighbors were determined by K-nearest neighbors and mutual nearest neighbors algorithms. Imputation was conducted by the TransferData function of Seurat using 50 dimensions and was applied only on the highly variable features of the scRNA-seq dataset retrieved by VariableFeatures function (number of features = 2000). Weight reduction was set to the previously computed LSI representation of the G4 scCUT&Tag module.

#### Integration using scBridge

For scBridge, AnnData objects were created using the Scanpy Python package (v1.9.8) [55], derived from the gene activity matrix of scCUT&Tag and the normalized count matrix of the corresponding scRNA-seq dataset (*Bartosovic *et al*.* for sorted data and *Zeisel *et al*.* scRNA-seq for unsorted data). Then, the objects were converted to.h5ad files. Only those genes were kept that can be found in both assays. The.h5ad files were fed into the scBridge pipeline. We visualized the joint embedding by UMAP. The scBridge experiment was conducted on Rocky Linux 8 OS using 4 × NVIDIA Tesla T4 GPU with CUDA 12.4.

#### Mapping query cells to reference

The unsorted G4 scCUT&Tag data were projected onto the sorted UMAP using the MapQuery wrapper function in Seurat. Anchor sets were determined with the FindTransferAnchors function, using CCA reduction with prioritizing gene activity assays. For the visualizations both the reference.reduction and reduction.model parameters were set to ‘umap’. The mapping efficiency was quantified by the corresponding prediction scores of the TransferData function.

### Visualization and quantification of integration efficiency

#### Prediction score of Seurat

Following classification of cells between modalities based on the scRNA-seq annotations, TransferData returns a prediction score assay. The calculation of prediction scores is detailed in *Stuart *et al. [43]. This metric was used to quantify the label transferring efficiency across the different clusters.

#### Overlap score

To quantify the similarity between cell clusters from the two modalities, we calculated an overlapping score as the sum of the minimum proportion of cells in each cluster that overlapped within each co-embedding cluster [34] [36]. Cluster overlaps varied from 0 to 1 and were visualized as a heatmap.

#### Reliability score of scBridge

The reliability score calculation and the reliability modeling of the cells are detailed in the original scBridge paper [35].

### Statistics

Plot statistics were calculated using the R package ggpubr (v0.6.0). The exact statistical tests are indicated in the figure legends. Significance of marker regions reported was tested using logistic regression with likelihood ratio test implemented in package Seurat with function FindAllMarkers. Cicero predictions with an override cutoff > 0.2 were selected for downstream analysis. For the quantification of genomic overlaps, GenomicRange and RegioneR [56, 57] was used. Venn diagrams were calculated and visualized by the eulerr package (https://CRAN.R-project.org/package=eulerr).

### AI

OpenCode (https://opencode.ai/BigPickle) was used to refactor and annotate the original code into the final by-figure organization, extend and modify existing analysis scripts, create download scripts for published datasets. All AI-generated code and content was read and scrutinized by the authors.

## Supplementary Information

Additional file1: Supplemental Figures S1–S7 and their legends. Figures S1–S7 presenting supplementary experimental results and analyses supporting the main figures

## Data Availability

The G4 scCUT&Tag data generated for this study have been deposited at the Gene Expression Omnibus under accession code: GSE291468 [58]. Datasets from prior studies that were analyzed here: GSE163532 (GFP + postnatal mouse brain P21 scRNA-seq) [30, 59], GSE151058 (mESC ATAC-Seq) [60]. Li et al. adult mouse brain cCREs are available here: http://catlas.org/mousebrain [36, 61]. Neuronal scRNA-seq data (level 2, loom files) were derived from the Mouse Brain Atlas of the Sten Linnarsson lab (http://mousebrain.org/) [33, 62]. Code to reproduce all figures is provided on GitHub and Zenodo (https://doi.org/10.5281/zenodo.23862054) [63, 64] under Creative Commons Attribution 4.0 International.
