# Supplementary material for: G-quadruplex profiling using single-cell CUT&Tag offers insight into the epigenetic landscape of the developing mouse brain
Source: Genome Biol. 2026 Nov 13;27:359. doi: 10.1186/s13059-026-04339-w (PMC13761071; doi:10.1186/s13059-026-04339-w)
Supplement: Supplementary file 1 [file 13059_2026_4339_MOESM1_ESM.pdf]

G-quadruplex profiling using single-cell CUT&Tag offers insight into the epigenetic landscape of the developing mouse brain

Jing Lyu[1,2,3,4], Szabolcs Hetey[1,2], Mehmet Ali Öztürk[3,4], Goncalo Castelo-Branco[2,5], Marek Bartosovic[5,6], Simon J Elsässer[1,2,3,4]

SUPPLEMENTAL INFORMATION

7 Supplemental Figures

SUPPLEMENTAL FIGURES

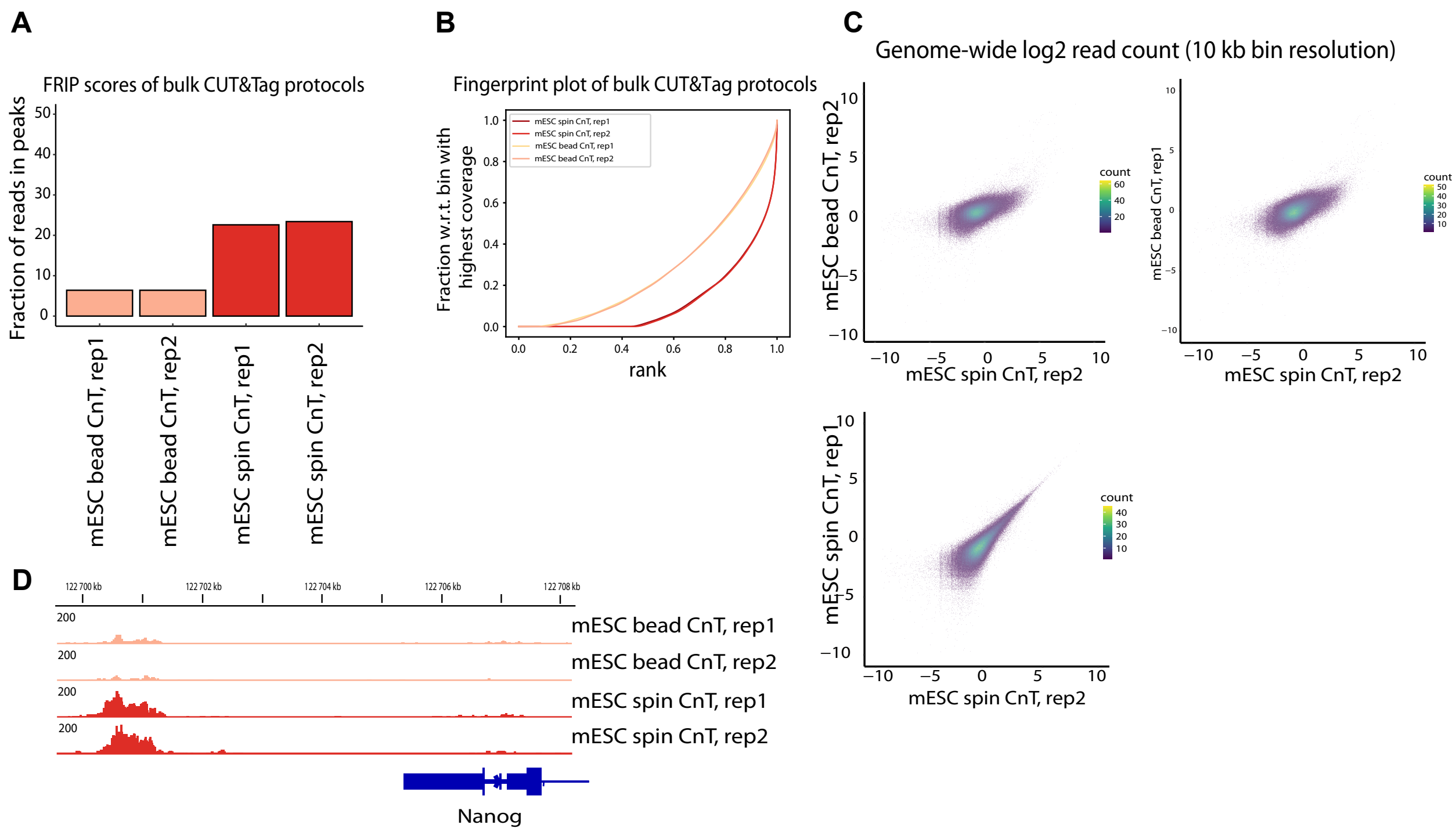

Fig. S1: Spin-based G4 CUT&Tag outperforms bead-based protocol
A) FRiP scores of bead- and spin-based bulk CUT&Tag protocols. **B**) Fingerprint plot (computed using deepTools) showing cumulative read coverage differences between the two bulk CUT&Tag protocols. **C**) Distribution of $\log_2$ read coverage across 10 kb genomic windows. **D**) Representative G4 landscape near the *Nanog* gene, illustrating signal-to-noise differences between bead- and spin-based protocols. mESC cluster-specific G4 peak over the gene *Lin28a*.

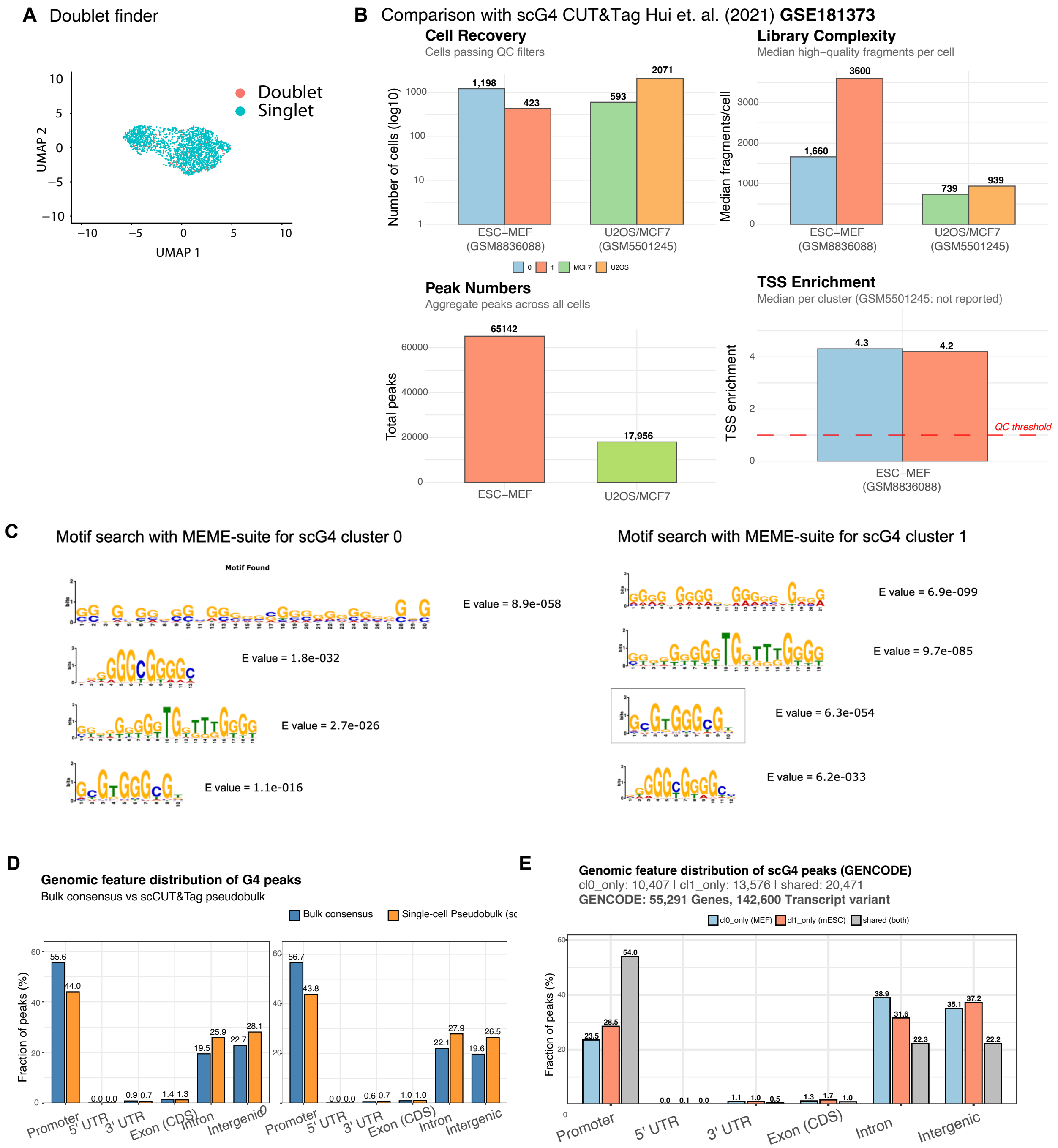

Fig. S2: Establishing G4 scCUT&Tag (scG4) with mixed mESC-MEF cell population. A.) UMAP of mESC-MEF G4 scCUT&Tag with the indication of identified doublets (by package DoubletFinder). **B.)** Quality comparison with Hui et. al. 2021 G4 scCUT&Tag experiment using mixed cancer cell populations. **C.)** Motif analysis (MEME-suite MEME-ChIP) of cluster 0 and cluster 1 peaks using default parameters. **D.)** Genomic distribution of scG4 and bulk G4 CUT&Tag peaks with respect to GENCODE annotation features: cluster1 vs bulk mESC, **E.)** Genomic distribution of scG4 and bulk G4 CUT&Tag peaks with respect to GENCODE annotation features: cluster 0 vs bulk MEF

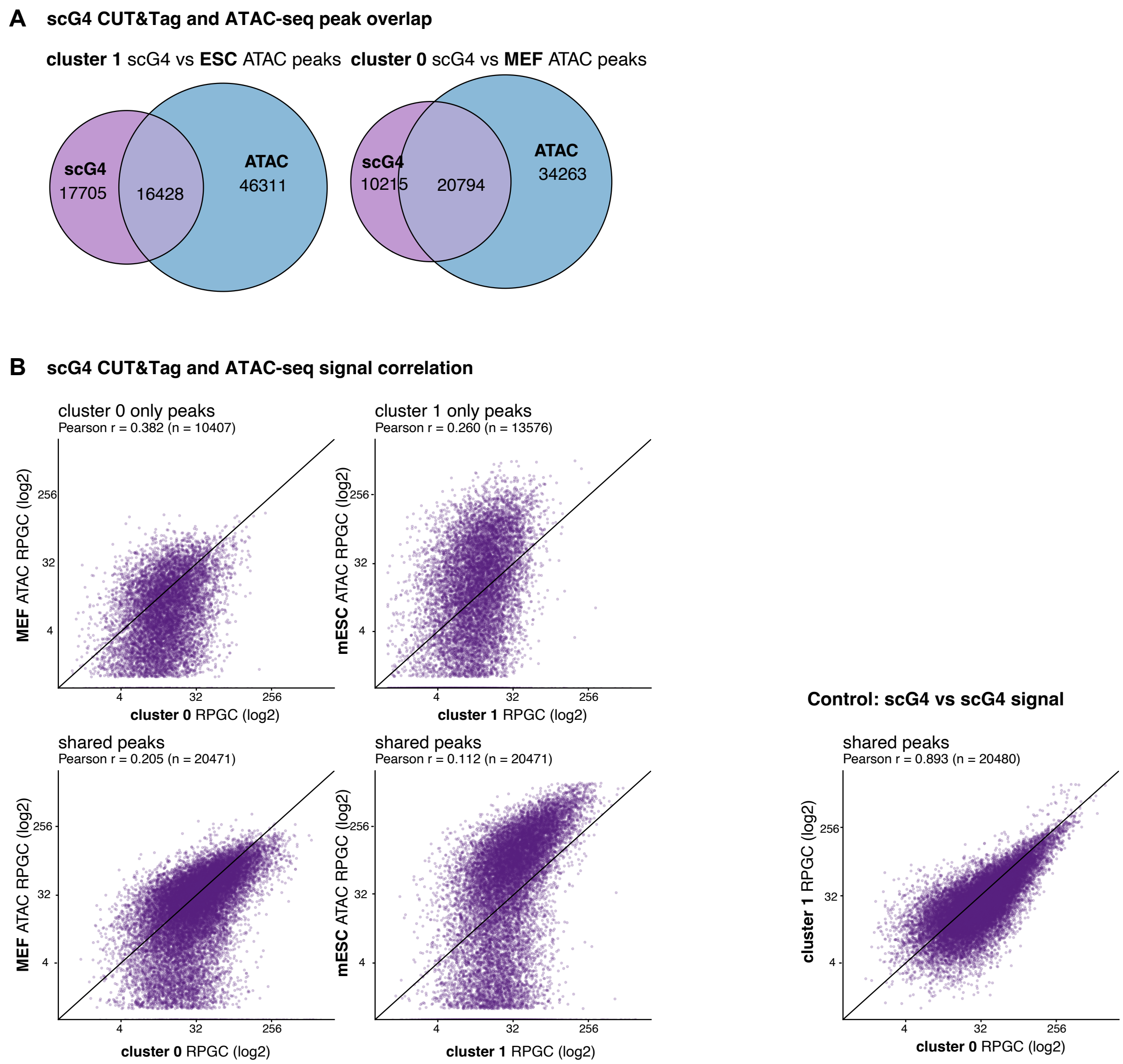

Fig. S3: Limited overlap and correlation between scG4 CUT&Tag peaks and ATAC-seq of the respective cell type . A) Venn diagrams between scG4 peaks per cluster and ATAC-seq peaks of the respective cell line. **B**) Related to Fig 1I: Top row from left: Pearson correlation between scG4 pseudobulk scG4 signal cluster 0 and MEF ATAC-seq signal at cluster 0-only G4 peaks (same as Fig 1I top); scG4 signal cluster 1 and mESC ATAC-seq signal at cluster 0-only G4 peaks (same as Fig 1I bottom). Bottom row from left: Pearson correlation between scG4 pseudobulk scG4 signal cluster 0 and MEF ATAC-seq signal at shared cluster0/1 G4 peaks; scG4 signal cluster 1 and mESC ATAC-seq signal at shared cluster0/1 G4 peaks; Pearson correlation between scG4 pseudobulk scG4 signal cluster 0 and scG4 pseudobulk scG4 signal cluster 0 at shared cluster0/1 G4 peaks

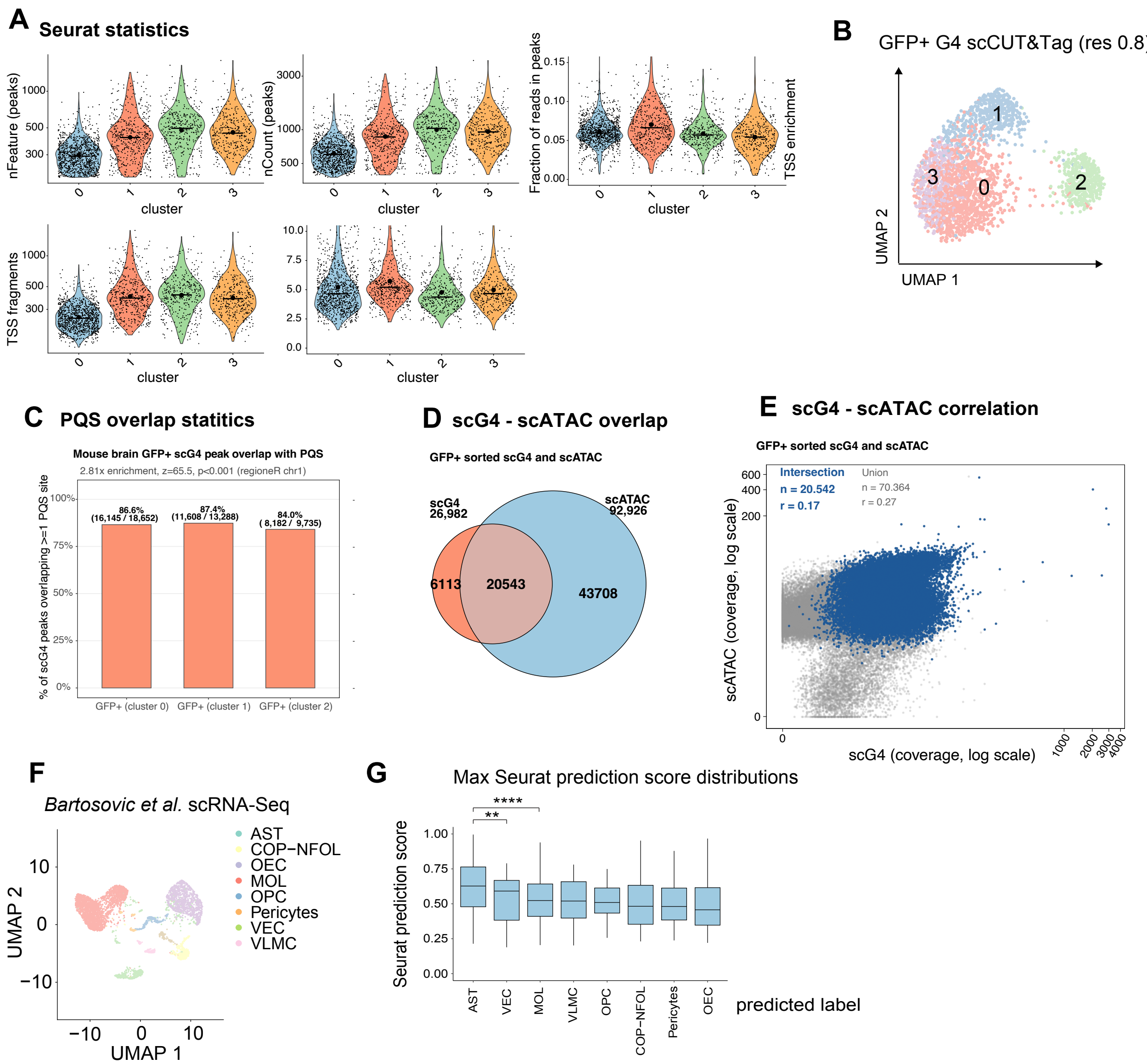

Fig. S4: Quality control, clustering and annotation of G4 CUT&Tag data from P21 mouse brain Sox10-GFP+ cells

A.) Violin plots showing the quality measures computed by Seurat. All the metrics were computed by Signac. **B**.) UMAP representation of the GFP+ sorted G4 scCUT&Tag before the integration made by CCA (Seurat). For clustering resolution parameter was set to 0.8 with the SLM algorithm. **C**.) Bargraph showing PQS overlap with scG4 peaks per cluster, results of regioneR permutation test is given for combined peaks. **D**.) Venn diagram showing overlap of scG4 and scATAC peaks. **E**.) Correlation between scG4 and matched scATAC signal for scG4-scATAC joint peaks and all peaks. **F**.) UMAP of the labeled, postnatal mouse brain scRNA-Seq dataset before the CCA. **G**.) Distributions of the maximum Seurat prediction scores per cell across the different cell types. P-values are calculated from two-tailed, Wilcoxon rank-sum tests.

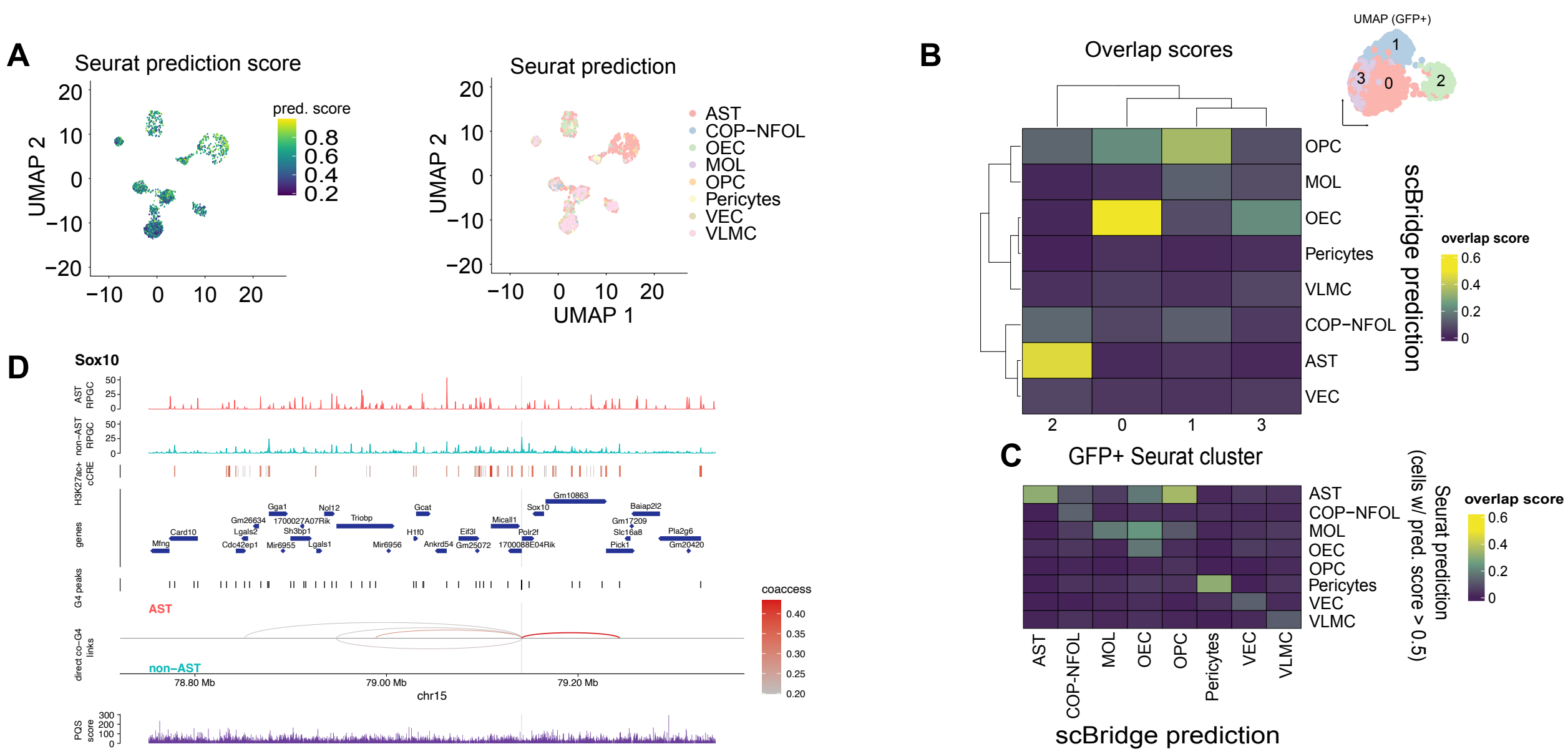

Fig. S5: scBridge clustering of G4 CUT&Tag and scRNA-Seq data

A.) UMAP plot of the joint embedding of scRNA-eq and G4 scCUT&Tag obtained by scBridge. Colors indicate the Seurat prediction scores and the predicted labels, respectively. **B**.) Heatmaps representing the overlap score quantifying the similarity upon label transfer between the labels predicted by scBridge and GFP+ G4 scCUT&Tag clusters. The UMAP represents the corresponding Seurat clusters. **C**.) Heatmap representing the overlap score quantifying the similarity upon label transfer between the scBridge and Seurat predictions. For the labels coming from the Seurat prediction only the cells with prediction score > 0.5 were considered. **D**.) Additional example to Figure 3I. Co-incidence analysis of G4 signals in single cells identifies examples of strong links between promoters and distal G4 sites in a cell-type-specific manner. Coverage plots show normalized G4 signal in AST (red) and non-AST (cyan) subpopulations. scG4 peaks and cCREs[38] are indicated. Cicero links (score > 0.2) are shown for AST and non-AST populations.

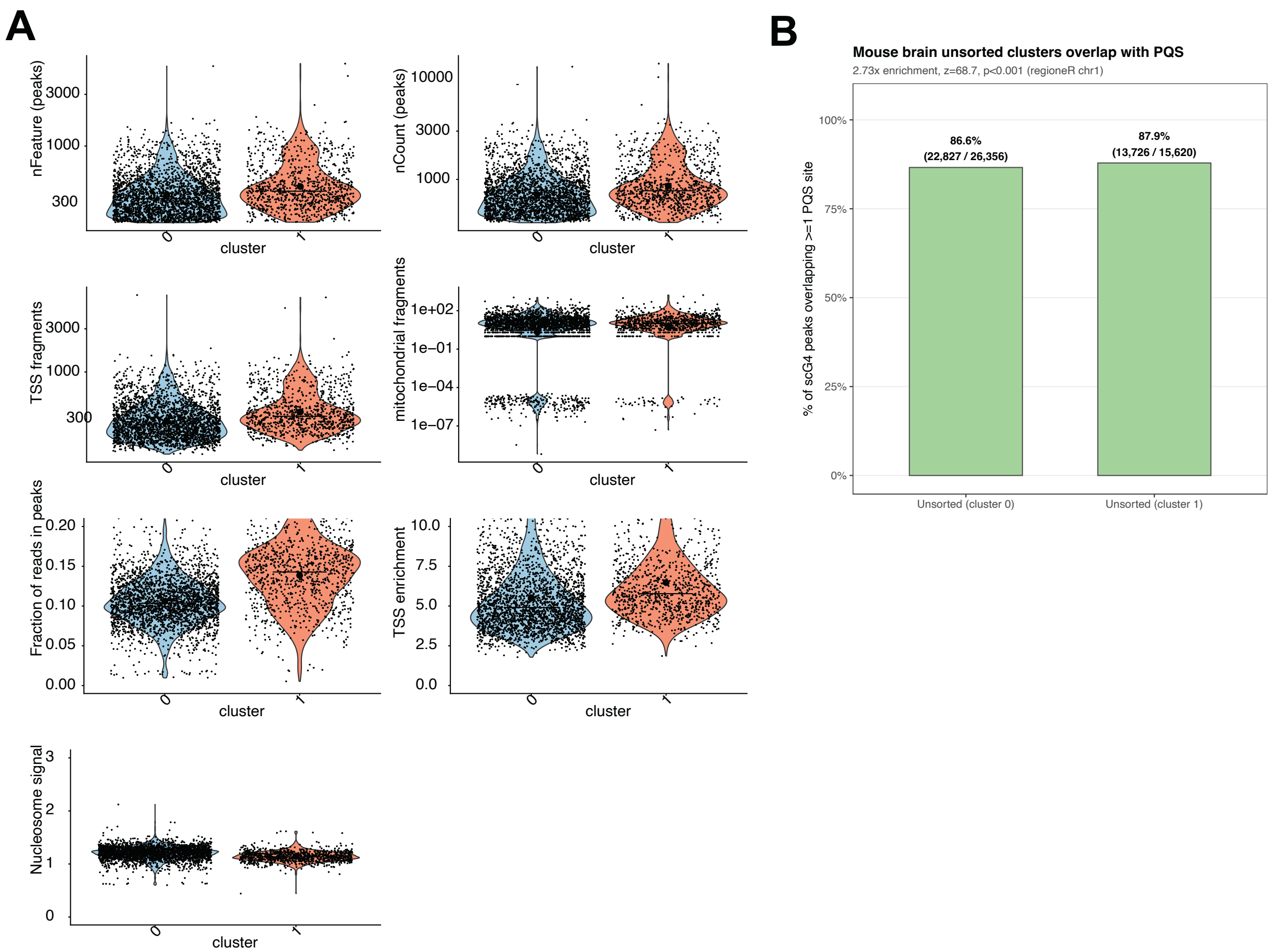

Fig. S6: Quality control of P21 mouse brain unsorted population scG4 CUT&Tag. A.) Violin plots showing the quality measures computed by Seurat. All the metrics were computed by Signac. **B**.) Bargraph showing PQS overlap with scG4 peaks per cluster, results of regioneR permutation test is given for combined peaks.

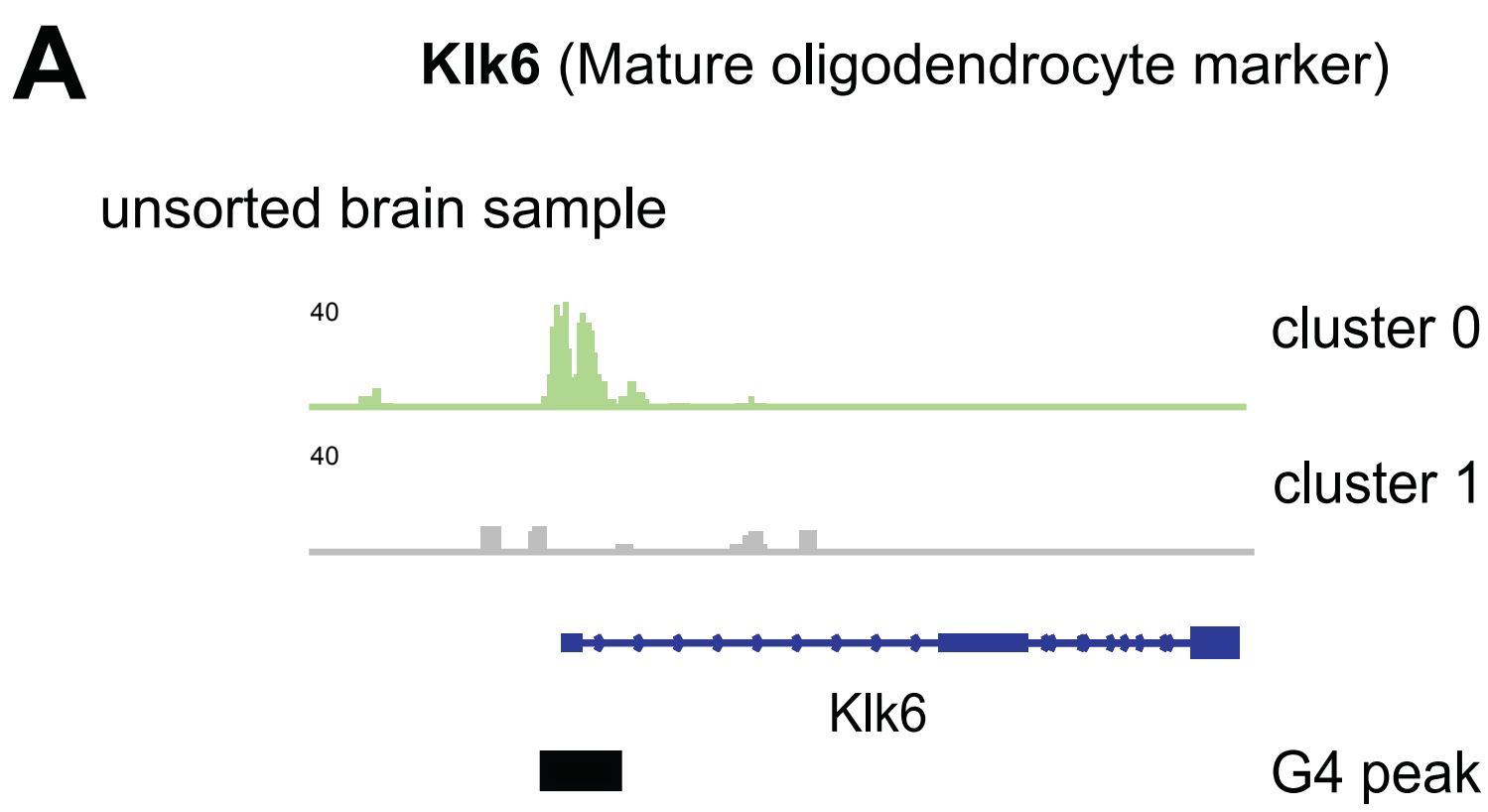

Fig. S7: Example oligodendrocyte marker in unsorted P21 clusters
A.) Violin plots showing the quality measures computed by Seurat. All the metrics were computed by Signac.
